# Supplementary material for: Comparative Analysis of Gut Microbiomes in Laboratory Chinchillas, Ferrets, and Marmots: Implications for Pathogen Infection Research
Source: Microorganisms. 2024 Mar 24;12(4):646. doi: 10.3390/microorganisms12040646 (PMC11051751; doi:10.3390/microorganisms12040646)
Supplement: Supplementary file 1 [file microorganisms-12-00646-s001.zip › supplementary materials/Supplementary figures.docx]

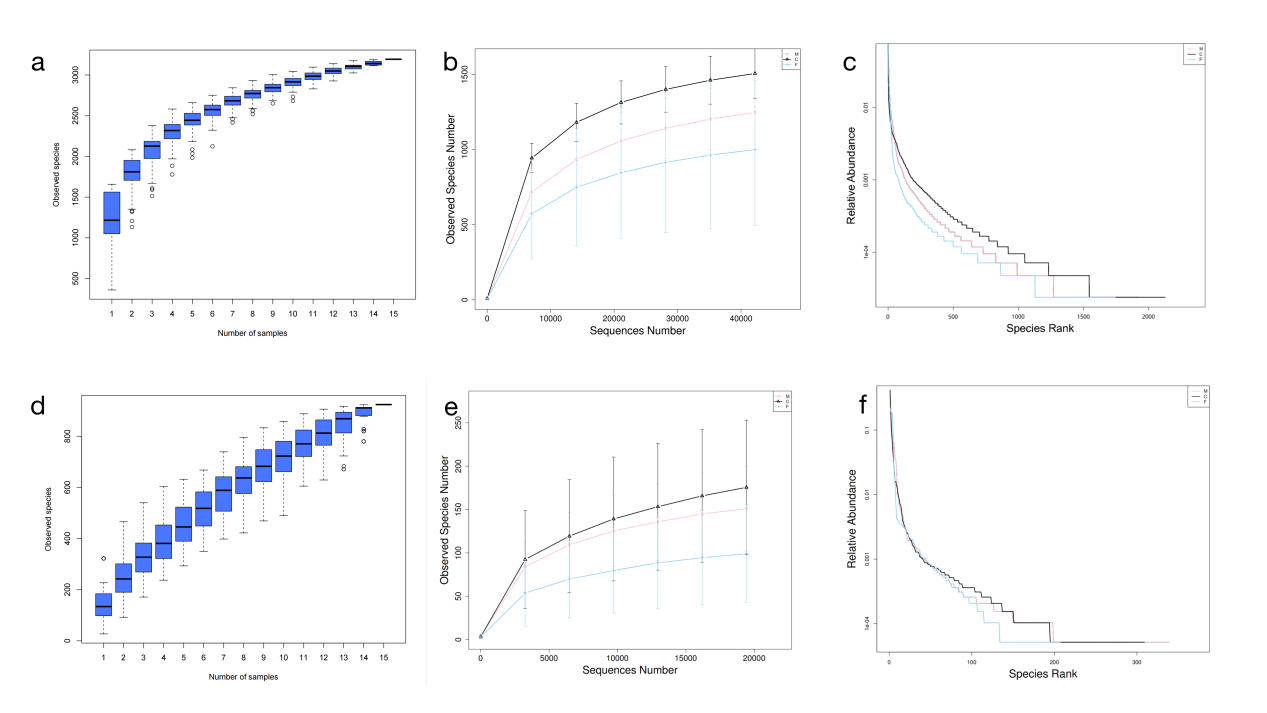


Figure S1 Species annotation results for bacteria (top) and fungi (bottom). (a,d) Species accumulation boxplots. (b,e) Species rarefraction curve. (d,f) Species rank abundance. Here, M,C, F represent the Marmot, Chinchilla and Ferret respectively.


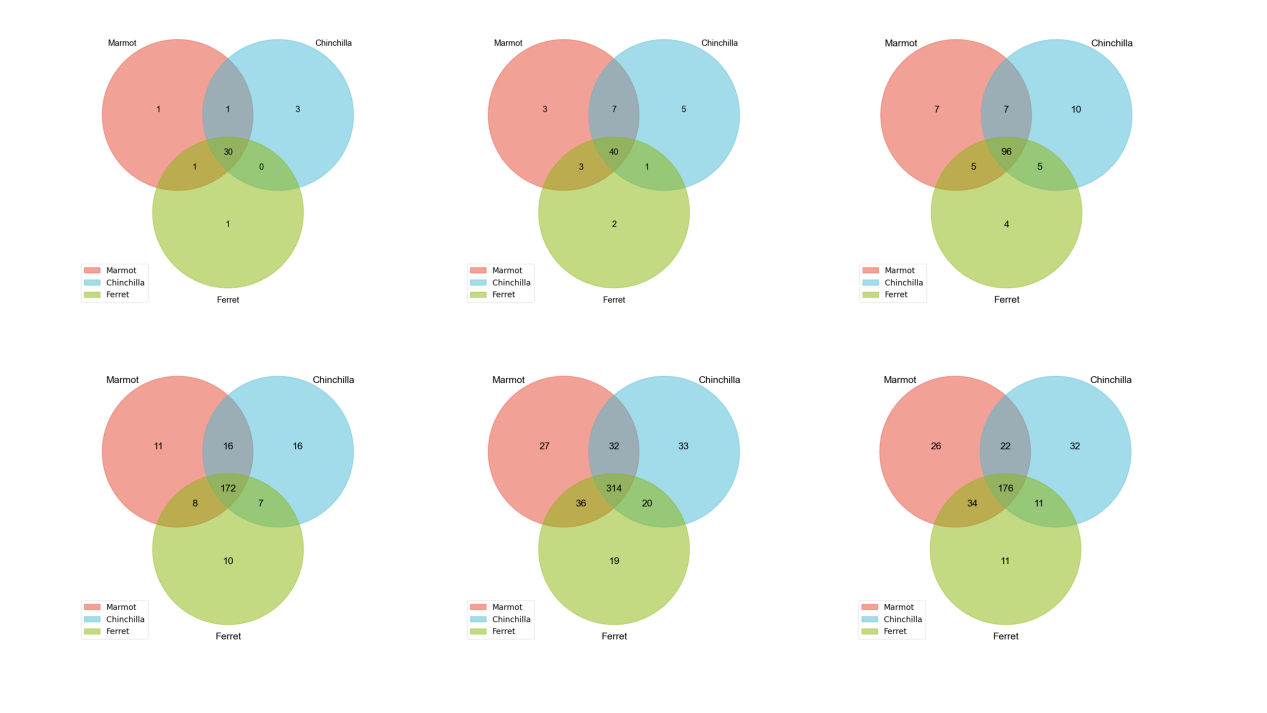


Figure S2 The distributions of bacteria annotations at the different levels (phylum, class, order, family, genus and species) for three animals.


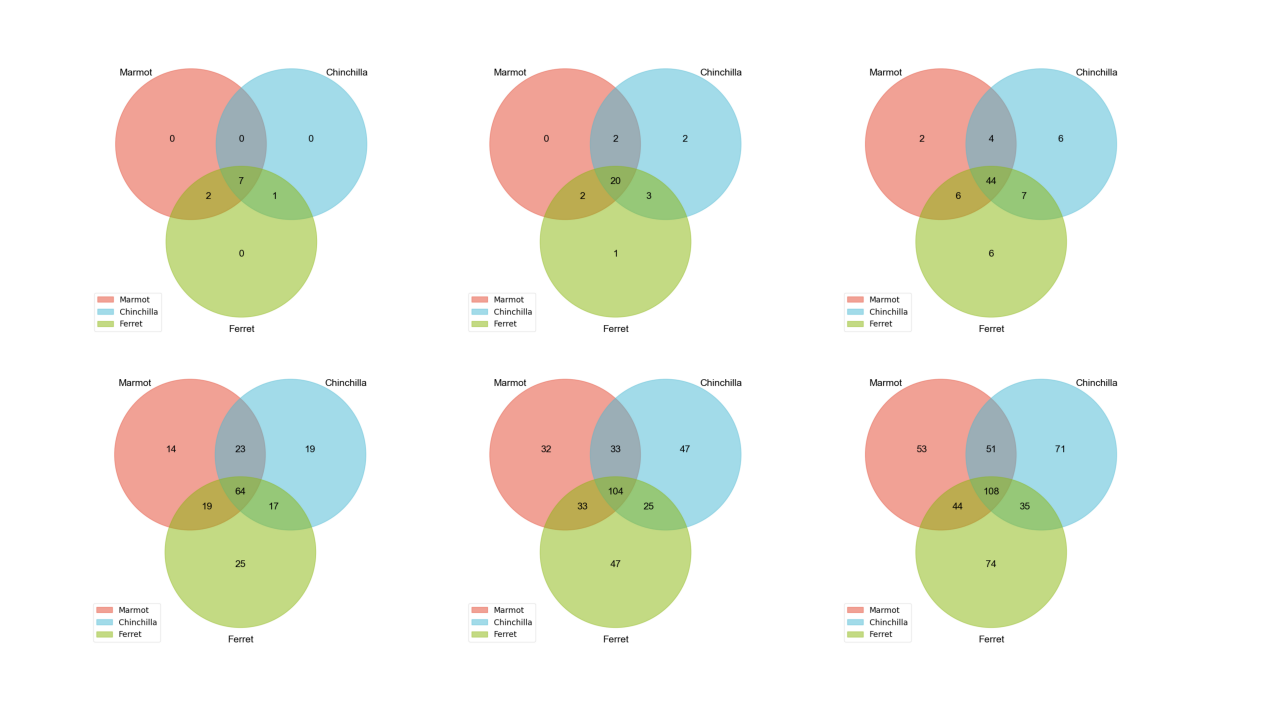


Figure S3 The distributions of fungi annotations at the different levels (phylum, class, order, family, genus and species) for three animals.


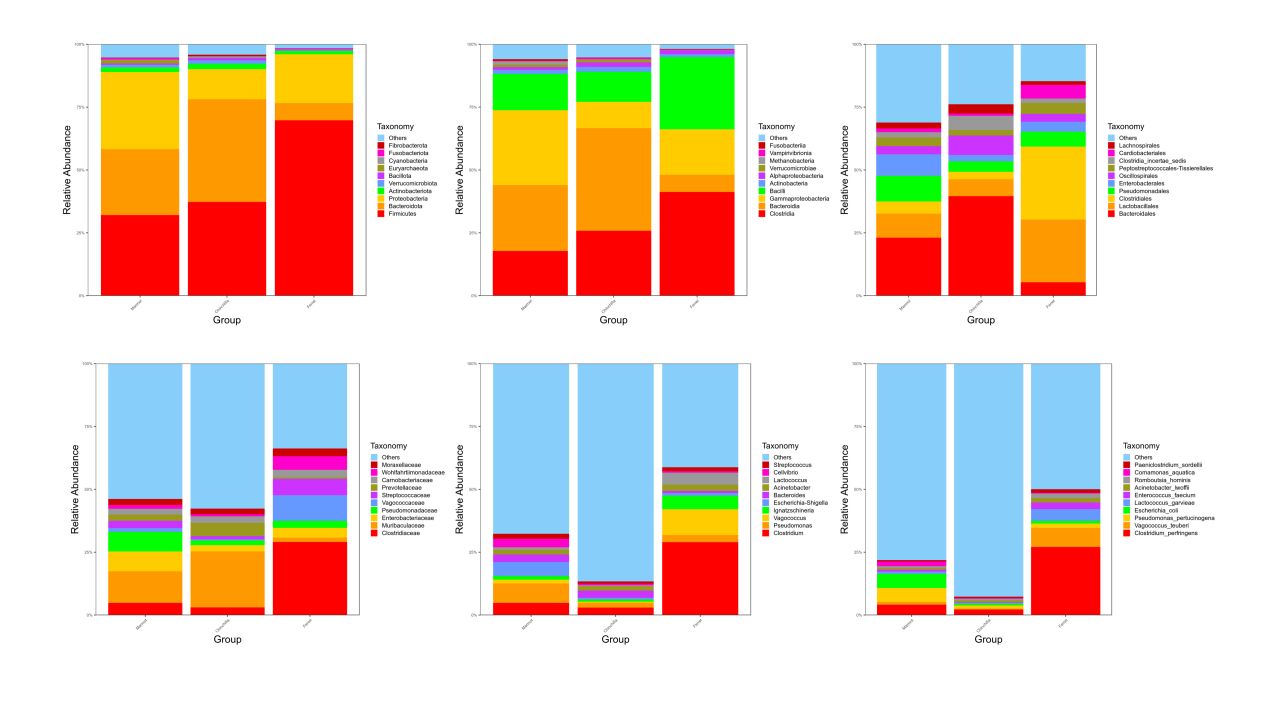


Figure S4 The top10 microbial composition of animals at different levels. The microbial composition at the kingdom, phylum, class, order, family and species level respectively. The horizontal axis is the name of three animals, and the vertical axis is the relatively abundant.


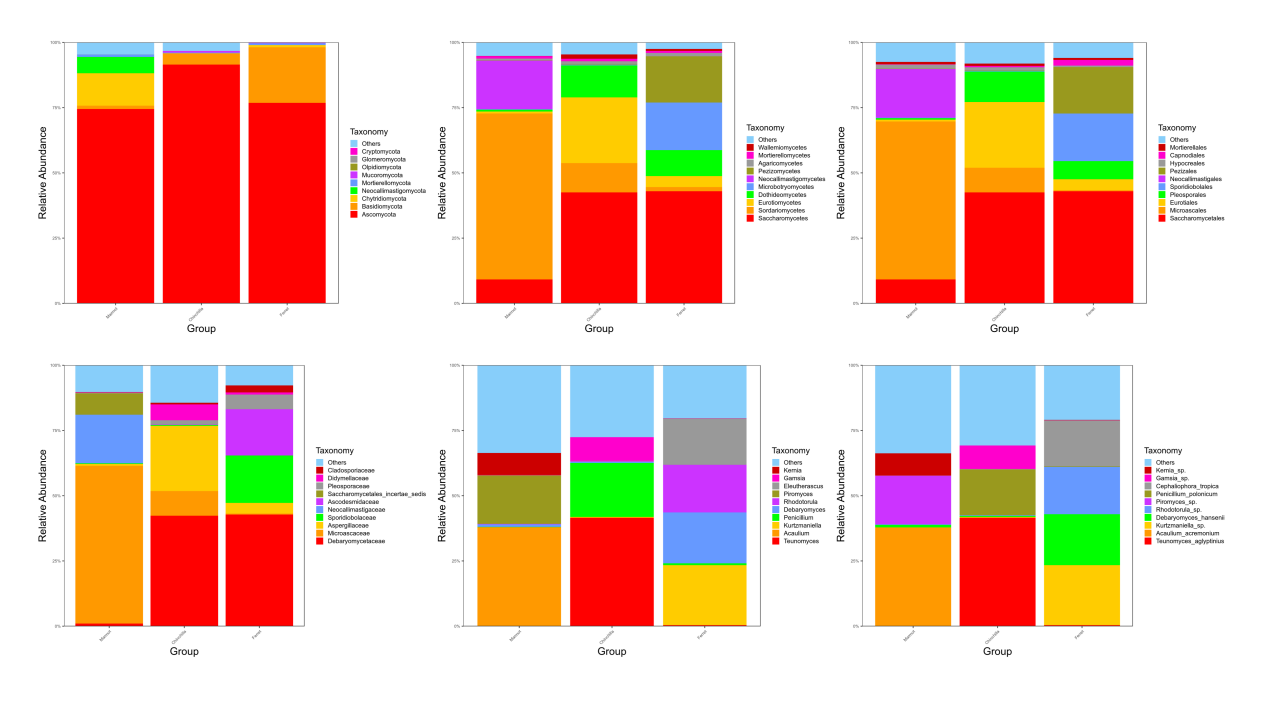


Figure S5 The top10 fungal composition of animals at different levels. The microbial composition at the kingdom, phylum, class, order, family and species level respectively. The horizontal axis is the name of three animals, and the vertical axis is the relatively abundant of microbes.


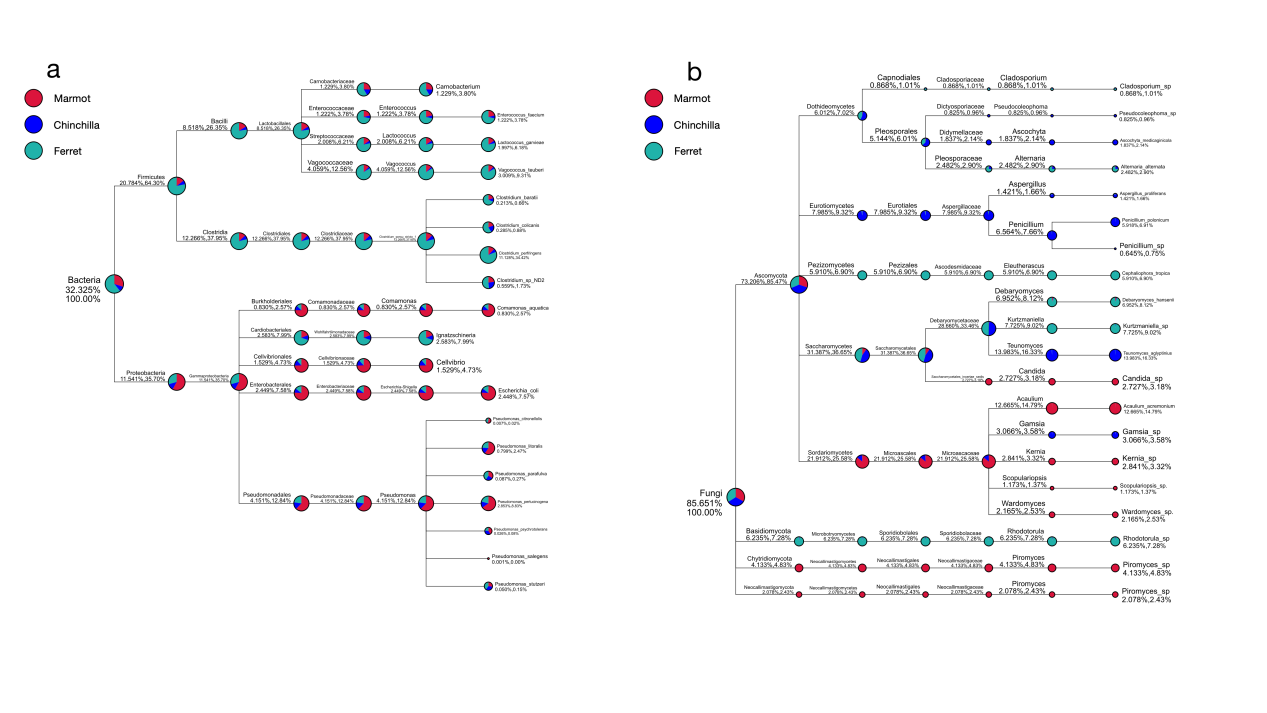


Figure S6 The distribution of the dominant microbes at the different levels for bacteria (a) and fungi (b). The different colored sectors in the circle represent different groups, corresponding to the legend on the left. The size of the sector represents the proportion of relative abundance of the group in that classification. The number below the classification name represents the average relative abundance percentage of all groups in that classification. There are two numbers, the former represents the percentage of all species, and the latter represents the percentage of the selected species.


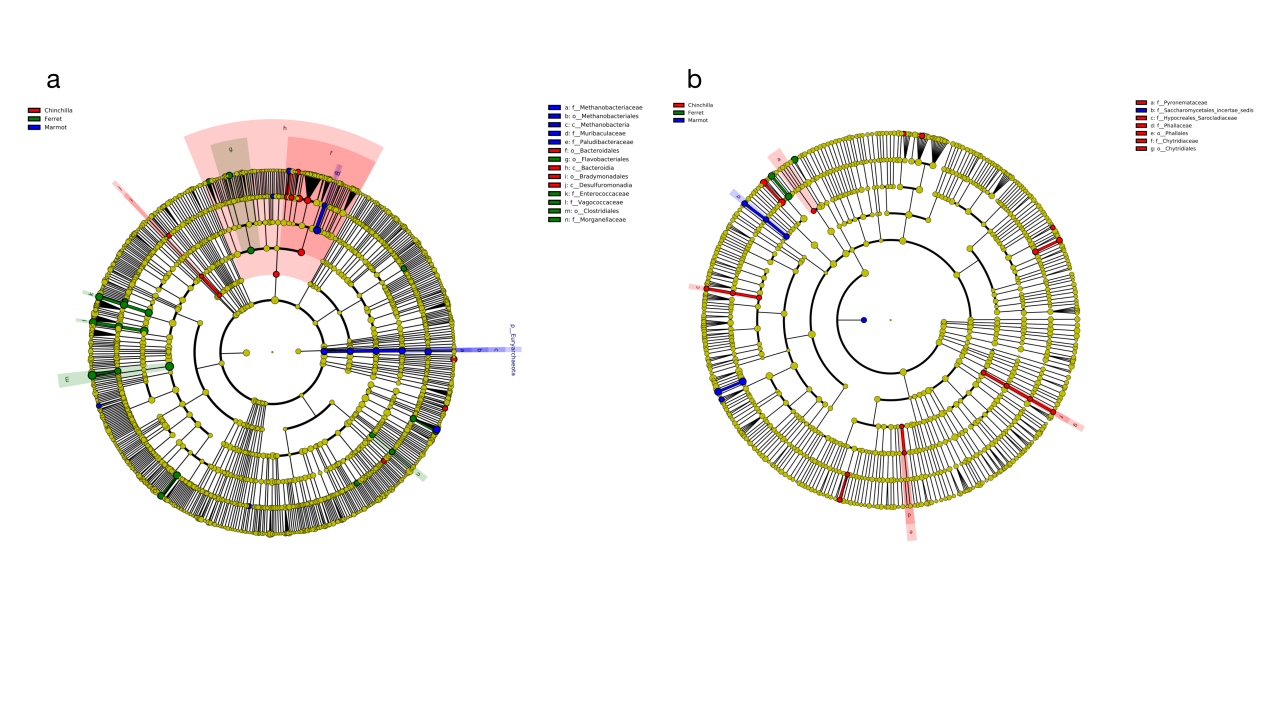


Figure S7 The LEfSe analysis branch diagram of animals for bacteria (a) and fungi (b). The node size corresponds to the average relative abundance of the taxa, and the hollow nodes represent taxa with insignificant differences between groups. The letters identify the names of taxa that differ significantly between the groups.


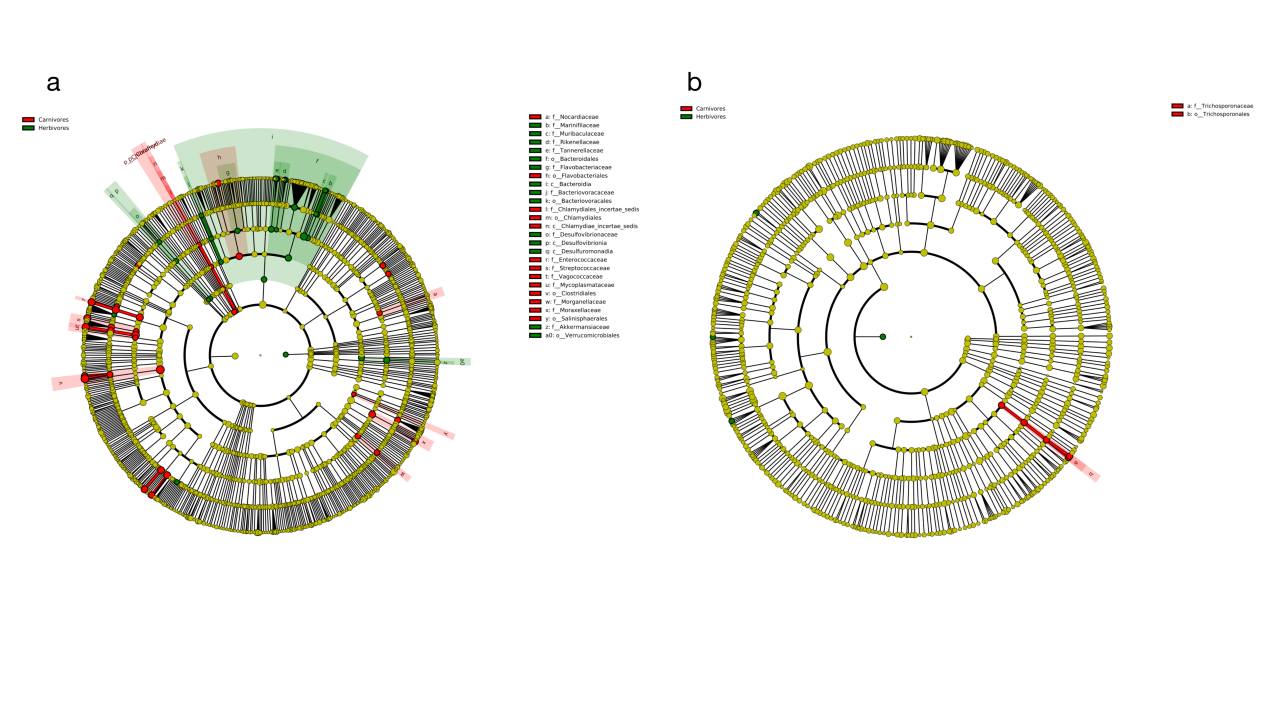


Figure S8 The LEfSe analysis branch diagram of feeding habitat for bacteria (a) and fungi (b). The node size corresponds to the average relative abundance of the taxa, and the hollow nodes represent taxa with insignificant differences between groups. The letters identify the names of taxa that differ significantly between the groups.


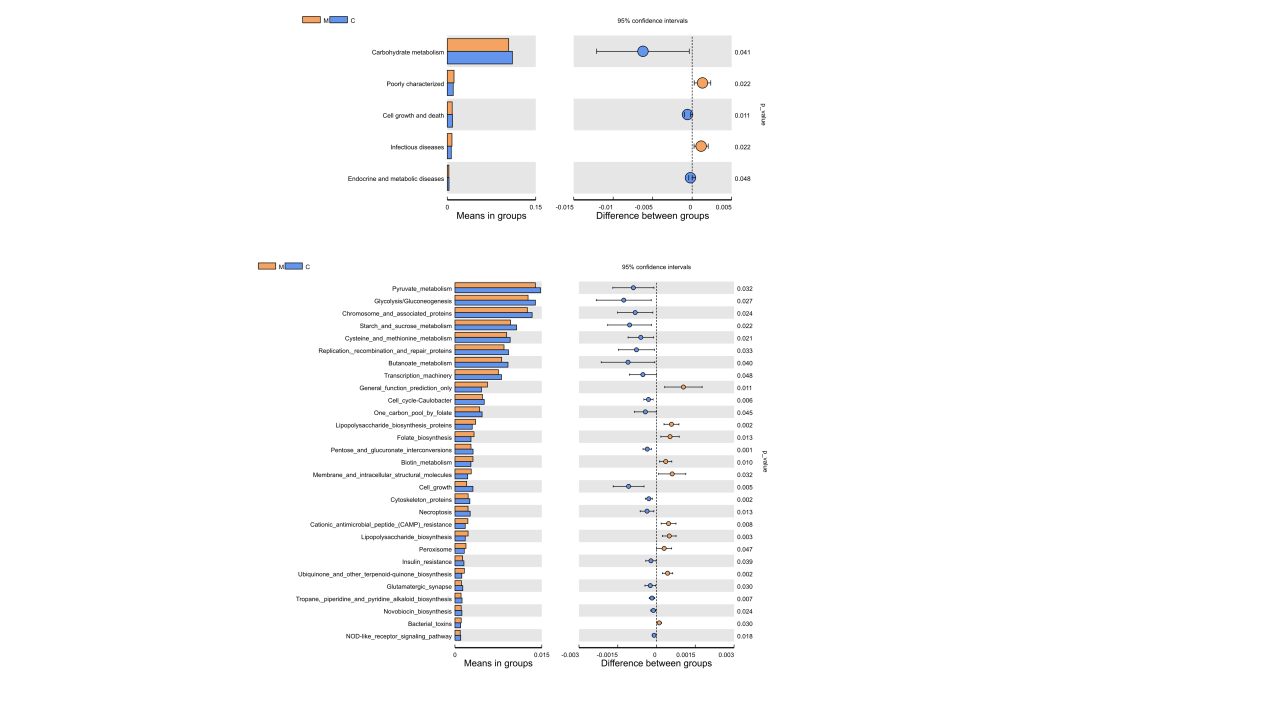


Figure S9 The different predictive functions between chinchilla and marmot.


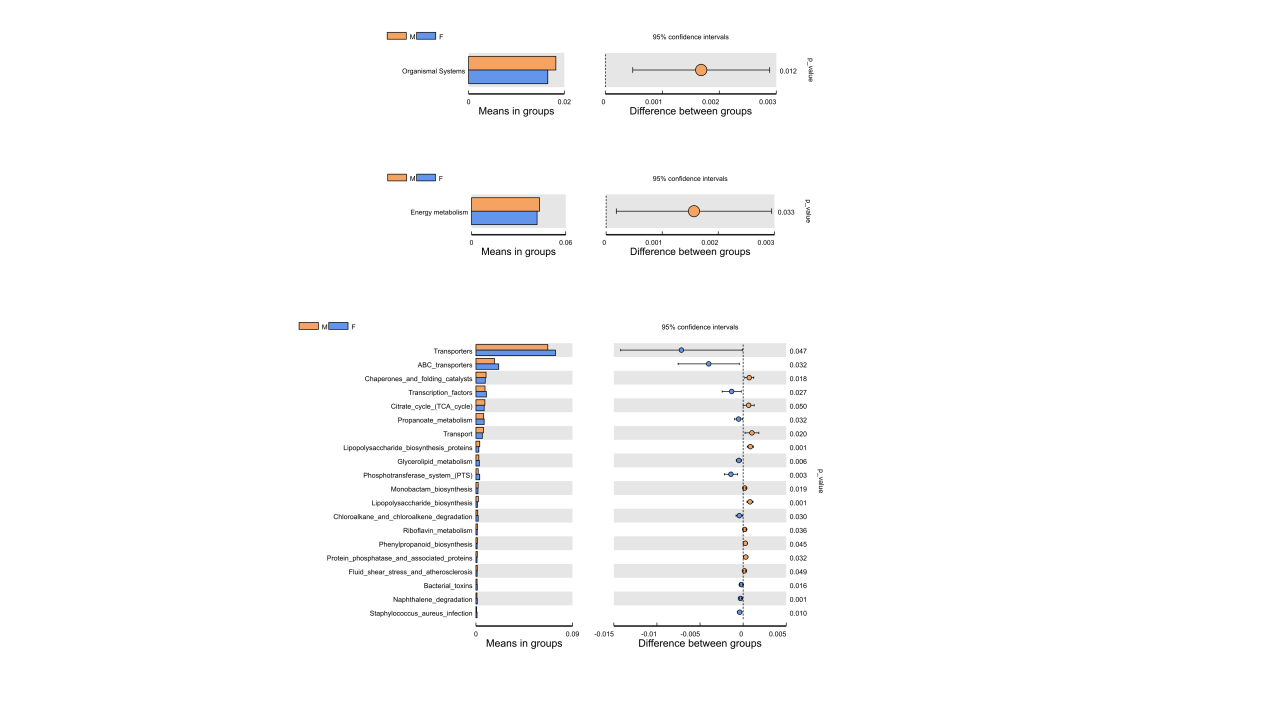


Figure S10 The different predictive functions between marmot and ferret.


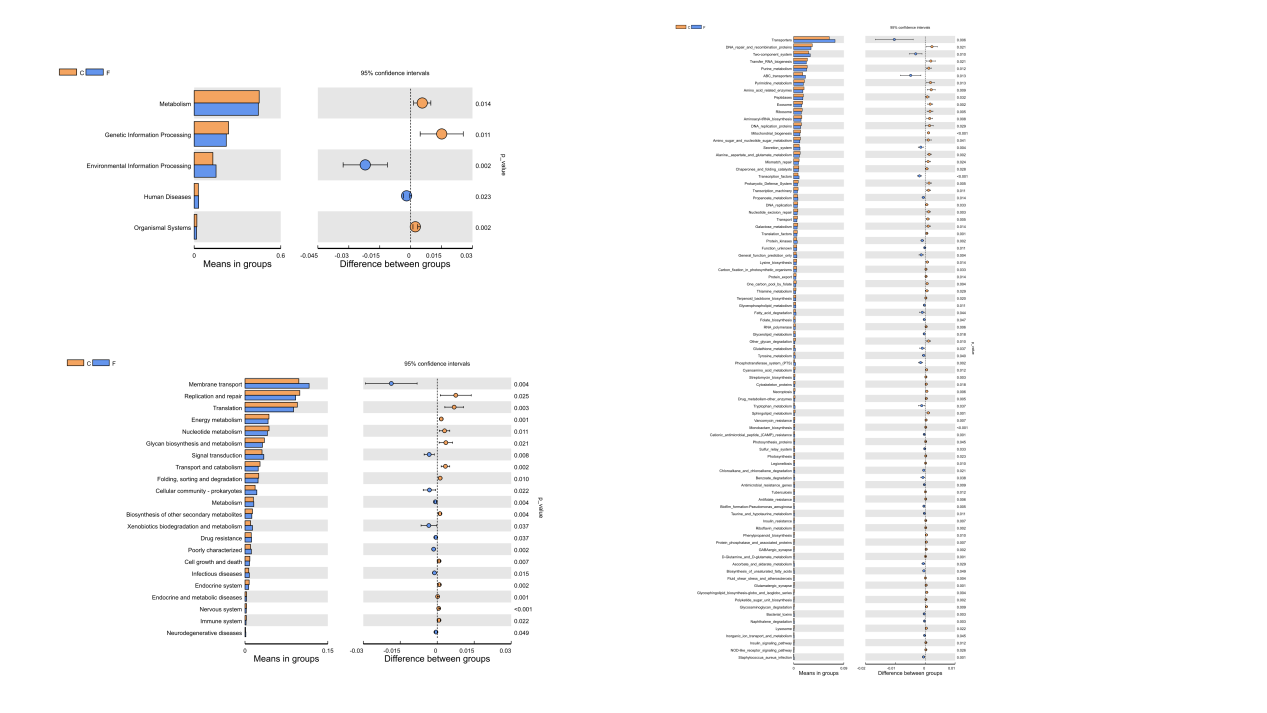


Figure S11 The different predictive functions between chinchilla and ferret.
