# Supplementary material for: Comparative Analysis of Gut Microbiomes in Laboratory Chinchillas, Ferrets, and Marmots: Implications for Pathogen Infection Research
Source: Microorganisms. 2024 Mar 24;12(4):646. doi: 10.3390/microorganisms12040646 (PMC11051751; doi:10.3390/microorganisms12040646)
Supplement: Supplementary file 1 [file microorganisms-12-00646-s001.zip › supplementary materials/Table S1.docx]

| **Table S1.** Fecal samples information from different animals. | | | | | | | |
| --- | --- | --- | --- | --- | --- | --- | --- |
| Species | Abbreviation | Sample name | Original sample number | Sample name in the report | Sex | Age/year | Samping date |
| Marmota monax | Marmot | TBS-7 | FZTD202317672-1A | M1 | male | 6 | 2020/6/30 |
| Marmota monax | Marmot | TBS-8 | FZTD202317673-1A | M2 | male | 5 | 2020/6/30 |
| Marmota monax | Marmot | TBS-9 | FZTD202317674-1A | M3 | male | 2 | 2020/6/30 |
| Marmota monax | Marmot | TBS-10 | FZTD202317675-1A | M4 | male | 2 | 2020/6/30 |
| Marmota monax | Marmot | TBS-11 | FZTD202317676-1A | M5 | male | 4 | 2020/6/30 |
| Chinchilla lanigera | Chinchilla | MSS-7 | FZTD202317677-1A | C1 | male | 2 | 2020/7/1 |
| Chinchilla lanigera | Chinchilla | MSS-8 | FZTD202317678-1A | C2 | male | 2 | 2020/7/1 |
| Chinchilla lanigera | Chinchilla | MSS-9 | FZTD202317679-1A | C3 | male | 1 | 2020/7/1 |
| Chinchilla lanigera | Chinchilla | MSS-10 | FZTD202317680-1A | C4 | male | 1 | 2020/7/1 |
| Chinchilla lanigera | Chinchilla | MSS-11 | FZTD202317681-1A | C5 | male | 2 | 2020/7/1 |
| Mustela putorius furo | Ferret | XD-7 | FZTD202317682-1A | F1 | male | 3 | 2020/6/30 |
| Mustela putorius furo | Ferret | XD-8 | FZTD202317683-1A | F2 | male | 2 | 2020/6/30 |
| Mustela putorius furo | Ferret | XD-9 | FZTD202317684-1A | F3 | male | 3 | 2020/6/30 |
| Mustela putorius furo | Ferret | XD-10 | FZTD202317685-1A | F4 | male | 4 | 2020/6/30 |
| Mustela putorius furo | Ferret | XD-11 | FZTD202317686-1A | F5 | male | 4 | 2020/6/30 |
