# Supplementary material for: Comparative Analysis of Gut Microbiomes in Laboratory Chinchillas, Ferrets, and Marmots: Implications for Pathogen Infection Research
Source: Microorganisms. 2024 Mar 24;12(4):646. doi: 10.3390/microorganisms12040646 (PMC11051751; doi:10.3390/microorganisms12040646)
Supplement: Supplementary file 1 [file microorganisms-12-00646-s001.zip › supplementary materials/Table S2.docx]

**Table S2.** Guaranteed value for product composition analysis of the chinchillas, marmots, and ferrets diets.

| Item | Chinchillas | Marmots | Ferrets |
| --- | --- | --- | --- |
| Guaranteed value |  |  |  |
| Crude protein | ≥170 g/kg | ≥200 g/kg | ≥34% |
| Crude fat | ≥30 g/kg | ≥30 g/kg | ≥16% |
| Crude fibre | 100～150 g/kg | 100～150 g/kg | ≤5% |
| Crude ash | ≤90 g/kg | ≤90 g/kg | ≤9% |
| Calcium | 10～15 g/kg | 10～15 g/kg | 1.4%～1.8% |
| Total phosphorus | 5～8 g/kg | 5～8 g/kg | 0.8%～1.4% |
| NaCl | ≥3 g/kg | ≥3 g/kg | 0.3%～1.0% |
| Moisture | ≤110 g/kg | ≤110 g/kg | ≤10% |
| Lysine | ≥8 g/kg | ≥8.5 g/kg | ≥1% |
| Vitamin A | ≥12500 IU/kg | ≥12500 IU/kg | ≥20000 IU/kg |
| Vitamin E | ≥70 IU/kg | ≥70 IU/kg | ≥120 IU/kg |
| Fe (Iron) | ≥150 mg | ≥150 mg | ≥338 mg/kg |
| Zn (Zinc) | ≥60 mg | ≥60 mg | ≥131 mg/kg |

Raw material composition of feed for Chinchillas: Corn, alfalfa, soybean meal, CaHPO_4_, NaCl, stone powder, vitamin premix, trace element premix, etc.

Raw material composition of feed for Marmots: Corn, soybean meal, alfalfa, CaHPO_4_, stone powder, NaCl, vitamin premix, trace element premix, etc.

Raw material composition of feed for Ferrets: American chicken meal, Peruvian fishmeal, Corn, soybean meal, wheat, CaHPO_4_, NaCl, DL-methionine, L-lysine, vitamin A, vitamin D_3_ and vitamin E, etc.
